# Identification of Mitral Annulus Hinge Point Based on Local Context Feature and Additive SVM Classifier

**DOI:** 10.1155/2015/419826

**Published:** 2015-05-18

**Authors:** Jianming Zhang, Yangchun Liu, Wei Xu

**Affiliations:** ^1^School of Computer and Communication Engineering, Changsha University of Science and Technology, Changsha 410114, China; ^2^School of Electronic Information and Electrical Engineering, Shanghai Jiao Tong University, Shanghai 200240, China

## Abstract

The position of the hinge point of mitral annulus (MA) is important for segmentation, modeling and multimodalities registration of cardiac structures. The main difficulties in identifying the hinge point of MA are the inherent noisy, low resolution of echocardiography, and so on. This work aims to automatically detect the hinge point of MA by combining local context feature with additive support vector machines (SVM) classifier. The innovations are as follows: (1) designing a local context feature for MA in cardiac ultrasound image; (2) applying the additive kernel SVM classifier to identify the candidates of the hinge point of MA; (3) designing a weighted density field of candidates which represents the blocks of candidates; and (4) estimating an adaptive threshold on the weighted density field to get the position of the hinge point of MA and exclude the error from SVM classifier. The proposed algorithm is tested on echocardiographic four-chamber image sequence of 10 pediatric patients. Compared with the manual selected hinge points of MA which are selected by professional doctors, the mean error is in 0.96 ± 1.04 mm. Additive SVM classifier can fast and accurately identify the MA hinge point.

## 1. Introduction

Congenital heart disease is one of the main reasons for death in children. The 3D shape and movement of the mitral apparatus are significant to analyze the function of left ventricular, diagnose mitral valve disease, and identify disorder of left ventricular [1–4]. There are some problems in surgical planning of mitral valve disease, including best surgical time and how to shape mitral valve. Therefore, analyzing the movement and shape of mitral apparatus with advanced computer technology and imaging technology has important clinical value and social value.

Precise positioning the hinge point of mitral value is helpful for modeling, motion tracking, and multimodalities registration of cardiac images. The vague and incomplete ventricular structure in ultrasound images due to the heavy noise, low resolution, and limited imaging range in real time echocardiography causes great difficulties to identify the mitral value manually and automatically. Nevo et al. [5] introduces an automated tracking algorithm using multidimensional dynamic programming which tracks the hinge point of mitral value leaflet in 2D echocardiographic images. Takemoto et al. [6] introduces an automated mitral annular tracking method. It adopts a partial shape constraint contour model to track and fit the ambiguous ventricular boundary and recognizes hinge point of mitral value using pattern matching algorithm. Veronesi et al. [7] tracks the mitral annular in 4D echocardiographic images combining optical flow method with block matching method. Due to the cumulative errors, the results require manual correction. Schneider et al. [8] use constrained optical flow combined with graph cut [9] and a valve state predictor to segment mitral annulus from four-dimensional ultrasound images.

This paper introduces a hinge point of mitral annulus identification algorithm based on additive SVM classifier [10, 11]. The optimized additive SVM classifier which can more quickly and efficiently classify test sample gets the same classification accuracy as classic SVM classifier. It is difficult to design a feature for echocardiographic image because of the heavy noise and low resolution. The typical global feature such as local binary pattern (LBP) [12, 13] and histogram cannot specify the hinge point in the whole cardiac structure. Spatial relationships of atrium and ventricle are fixed in the echocardiography. Therefore, in this work, a local context feature is obtained for subsequent classification of MA hinge point candidates for intracardiac structures in echocardiography. Ideally, every pixel in the neighborhood can be put into context. However, this would generate a large feature space. This paper designs a local context feature which sparely samples the gray value of the context locations on eight directions in 45-degree intervals. Reasonable results will be achieved after applying the additive SVM classifier on this local context feature. The rest of this paper is organized as follows.  Section 2 introduces additive SVM classifier. In Section 3, we describe the local context feature.  Section 4 gives an improved method. In Section 5, we present the flow of classification.  Section 6 shows some experimental results that demonstrate the effectiveness of this algorithm. Finally, we conclude in Section 7.

## 2. Additive SVM Classifier

SVM and boosted decision tree [14, 15] are the two main methods in target detection and multitargets recognition. Classifiers based on boosted decision trees have faster classification speed, but they are significantly slower to train. Furthermore, the complexity of training grows exponentially with the number of classes. The linear SVM is efficient during training on a given feature space. It can be used in real-time applications for low memory requirements and fast classification speed. Although the kernel trick is introduced to handle nonlinear problems in SVM, its complexity is much higher than linear SVM.

The linear SVM is more efficient, but many nonlinear kernels can get better results in pattern classification tasks due to the nonlinear distribution of features. Some popular nonlinear kernels which are based on histograms of low-level features like color and texture of the image use a kernel derived from histogram intersection or chi-squared distance to train a SVM classifier. To evaluate the classification function, the test histogram is compared with every support vector histogram. Maji et al. [10, 11] present a method which can efficiently compute the classification function based on histograms. This optimized method improves additive kernel SVMs significantly and can be used in any additive kernels.

### 2.1. Histogram Intersection Kernel SVMs

Given training set {(*y*_*i*_, **x**_*i*_)}_*i*=1_^*N*^, class label *y*_*i*_ ∈ {−1,1}; vector **x**_*i*_ ∈ **R** ^*n*^. To find the hyper plane to separate the sample set in linear problem, the minimization function can be written as

(1) $$\begin{array}{c} \phi \left(\;w,\xi \;\right)=\frac{1}{2}{\left\|\;w\;\right\|}^{2}+C\overset{N}{\underset{i=1}{\sum }}\xi_{i}, \end{array}$$

where

(2) $$\begin{array}{c} y_{i}\times \left(\;w\cdot x_{i}\;\right)+b\geq 1-\xi_{i},\;i=1,2,\ldots ,N, \end{array}$$

and *ξ*_*i*_ ≥ 0 is the slack variable, (1/2)‖**w**‖^2^ is used to maximize the distance between support vectors and hyper plane, *C*∑_*i*=1_^*N*^ *ξ*_*i*_ is used to minimize the error rate, *C* > 0 is the weight between maximized distance and slack constraints, **w** is the normal vector to the hyperplane, and *b* determines the offset of the hyperplane from the origin along the normal vector **w**. The kernel *K*(*x*, *z*) : *R* ^*n*^ × *R* ^*n*^ → *R* is the inner product *φ*(**x**) · *φ*(**z**) in high dimension. The maximized duality function is

(3) $$\begin{array}{c} W\left(\;\alpha \;\right)=\overset{N}{\underset{i=1}{\sum }}\alpha_{i}-\frac{1}{2}\overset{N}{\underset{i,j=1}{\sum }}\alpha_{i}\alpha_{j}y_{i}y_{j}K\left(\;x_{i},x_{j}\;\right), \end{array}$$

where *α* is Lagrange multiplier, 0 ≤ *α*_*i*_ ≤ *C*, ∑*α*_*i*_ *y*_*i*_ = 0. Decision function is sgn(*h*(*x*)), where

(4) $$\begin{array}{c} h\left(\;x\;\right)=\overset{m}{\underset{l=1}{\sum }}\alpha_{l}y_{l}K\left(\;x_{l},x\;\right)+b. \end{array}$$

And **x**_*l*_, *l* ∈ {1,2,…, *m*} is the support vector. Therefore, classifying a test feature requires *m* times calculation of kernel function and stores *m* support vectors. Assuming that the complexity of decision function is *O*(*n*), the complexity of classification of one test feature is *O*(*m*∗*n*). As to linear kernel function *K*(**x**, **z**) = **x** · **z**, its decision function is *h*(**x**) = **w** · **x** + *b* where **w** = ∑_*l*=1_^*m*^ *α*_*l*_ *y*_*l*_ **x**_*l*_. So the complexity of linear SVM is *O*(*n*).

With similarity of feature such as boundary, color can be represented as histogram which regularly uses histogram intersection as its evaluation of similarity. The histogram intersection kernel is

(5) $$\begin{array}{c} K_{\min}\left(\;x,z\;\right)=\overset{n}{\underset{i}{\sum }}\min\left(\;x_{i},z_{i}\;\right), \end{array}$$

where **x**, **z** ∈ **R**_+_^*n*^ is histogram feature. The decision function is

(6) hz∑l=1mαlylKmin⁡z,xl+b=∑l=1mαlyl∑i=1nmin⁡zi,xl,i+b.

The complexity of (6) is still *O*(*m*∗*n*). The key property of intersection kernels is that the order of summing can be exchanged. So, (6) can be converted to the following:

(7) hz∑l=1mαlyl∑i=1nmin⁡⁡zi,xl,i+b=∑i=1n∑l=1mαlylmin⁡⁡zi,xl,i+b=∑i=1nhizi+b.

Function *h*(·) can be represented as the sum of 1D functions *h*_*i*_(·), where

(8) $$\begin{array}{c} h_{i}\left(\;z_{i}\;\right)=\overset{m}{\underset{l=1}{\sum }}\alpha_{l}y_{l}\min\left(\;z_{i},x_{l,i}\;\right). \end{array}$$

So as to make the complexity of *h*_*i*_ to *O*(log⁡*m*), let ${\overset{-}{x}}_{l,i}$ denote the sorted *x*_*l*,*i*_ in increasing order; the corresponding *α*_*l*_ and *y*_*l*_ are ${\overset{-}{\alpha }}_{l}$ and ${\overset{-}{y}}_{l}$. If $z_{i}<{\overset{-}{x}}_{1,l}$, then $h_{i}\left(z_{i}\right)=z_{i}\sum_{l}{\bar{\alpha }}_{l}{\bar{y}}_{l}=0$. Otherwise, let *r* be the largest integer satisfying ${\overset{-}{x}}_{r,l}\leq z_{i}$. The function *h*_*i*_(·) is transformed to

(9) hizi∑l=1mα¯ly¯lmin⁡zi,xl,i=∑1≤l≤rα¯ly¯lxl,i+zi∑r<l≤mα¯ly¯l=Air+ziBir,

where $A_{i}\left(r\right)=\sum_{1\leq l\leq r}{\overset{-}{\alpha }}_{l}{\bar{y}}_{l}x_{l,i}$, $B_{i}\left(r\right)=\sum_{r<l\leq m}{\bar{\alpha }}_{l}{\bar{y}}_{l}$. Functions *A*_*i*_ and *B*_*i*_ which only depend on the support vectors and *α* can be computed after SVM model is trained. Binary search is adopted to get *r* and reduces the complexity.

## 3. Local Context Feature

The general image detection operator such as the Sobel operator and the Laplace operator cannot be applied to ultrasound images due to the heavy noise and blurred boundary. Context is the relationship with the neighbors which can be represented as a certain range of neighbors of a pixel in image processing. This paper introduces a local context feature which sparsely sample [16] the neighbor pixels of a pixel. Sampling sparsely can decrease the dimensions and shorten the training time. Because of the fixed spatial distribution of cardiac tissues in ultrasound images, the local context feature can quantify the position relationship between cardiac tissues.

**Figure 1:**
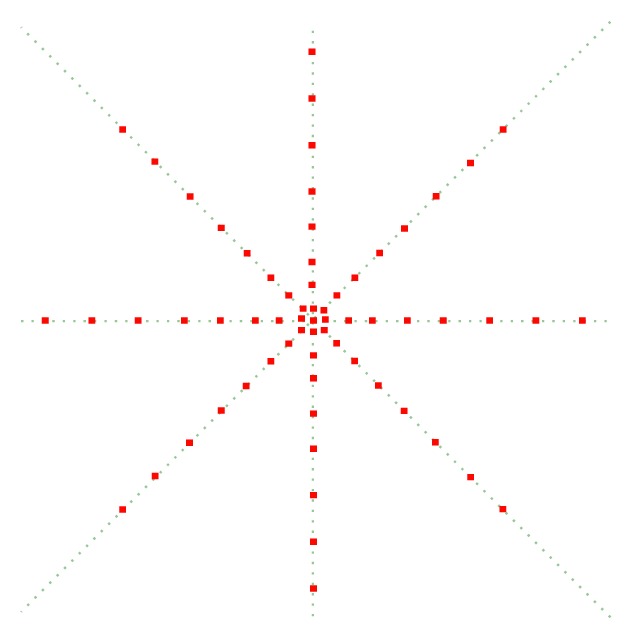
Local context feature.

As Figure 1 shows, red points represent the sampling points. This feature sampling at eight directions and the intervals between sample points largen as the distance to the center point lengthens. The points nearer to the center point contain more information about the center point, so most feature samples points are in near range. Taking the size of cardiac ultrasound image into account, the max sampling distance sets as 29 and the sampling position at each direction is {1,3, 5,9, 13,17,23,29}. Because of the heavy noise in echocardiography, the gray value of sampling point will make training and classification features inaccurate, which will reduce the recognition rate. In order to eliminate the error caused by the noise, we apply mean filtering to the sampling points when sampling. Because the larger average template cannot make the sampling gray value accurately reflect the information of sampling points, we set the average of 3 ∗ 3 neighborhoods as the value of each sampling point. So one pixel can get a 65-dimension feature and the local feature is extracted fast.

## 4. Refine the Classification

**Figure 2:**
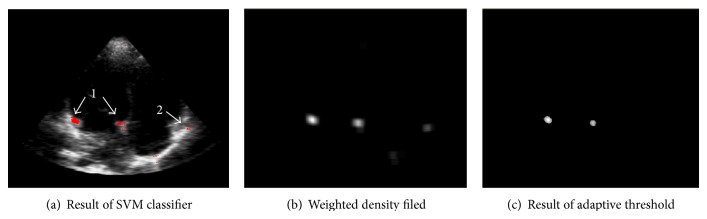
Classification result of additive SVM.

We can get a good recognition result by adopting the local context feature and additive kernel SVM classifier, as Figure 2(a) shows. Red points are the candidate points given by the SVM classifier. The points that arrow 1 indicates get correct classification result. Due to the low resolution and heavy noise, the point that the arrow 2 indicates gets a misclassification result by SVM.

**Figure 3:**
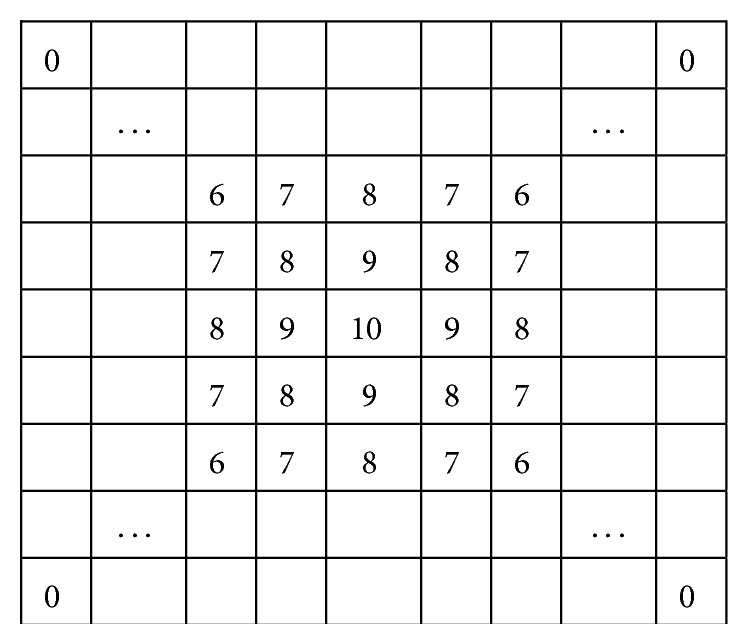
The weighted template obtained by block distance.

The SVM classifier is trained to get points like that arrow 1 indicates, so the majority of candidate points will be right. And density is a good feature to distinguish the right points and wrong ones. This paper applies a weighted template to each candidate point and gets a weighted density field.  Figure 3 shows the weighted template obtained by block distance.

The density field function is

(10) $$\begin{array}{c} F\left(\;A\;\right)=\underset{y\in C\cap N_{11}\left(A\right)}{\sum }\left[\;10-D_{\text{block}}\left(\;y,A\;\right)\;\right], \end{array}$$

where *A* is any point in density field, *C* is the set of candidate points, *D*_block_(·) is the block distance, *N*_11_(*A*) is the 11 neighborhoods of a point *A*. As Figure 2(c) shows, the right points are highlight in the density field and wrong points are dim. We can determine an adaptive threshold to exclude the dim points by binary search between the max and min values of density field. The following part shows the flow of the algorithm.

*The Flow of Adaptive Thresholding*. *H* = the max value in density field.

1. If the number of continuous areas greater than *H* is exact two, go to step (4).
2. If the number of areas is less than two, decrease *H* and go to step (1).
3. If the number of areas is greater than two, increase *H* and go to step (1).
4. Get the adaptive threshold *H*.

**Figure 4:**
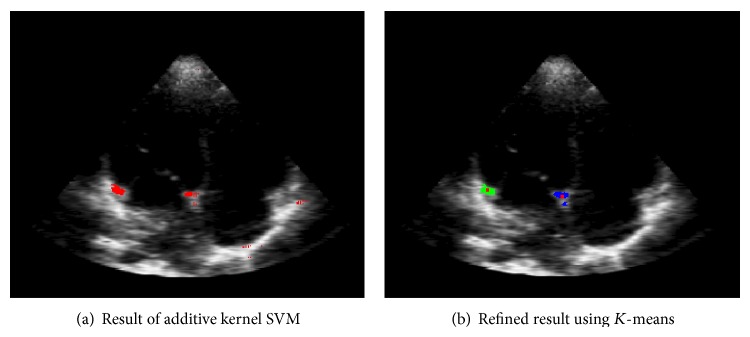
Refined classification result of *K*-means classifier.

Figure 2(c) shows the result adopting adaptive threshold; we can see that it is so easy to separate the two blocks. Then the *x* and *y* average values of the two blocks can be calculated separately as the position of hinge point of mitral annulus. Another problem is that the adaptive threshold may also exclude some right points which lead to an unreliable result. In order to get an accurate result, we propose to use the two blocks centers as the initial clustering center of *K*-means to classify the whole candidate points in a certain scale. Due to the fixed size of mitral annulus, the certain scale can be obtained from experiments. The result is shown in Figure 4, the green and blue block are the results of *K*-means classifier, and the center red points in the two blocks are the accurate position of hinge point of mitral annulus.

## 5. Classification Flow

**Figure 5:**
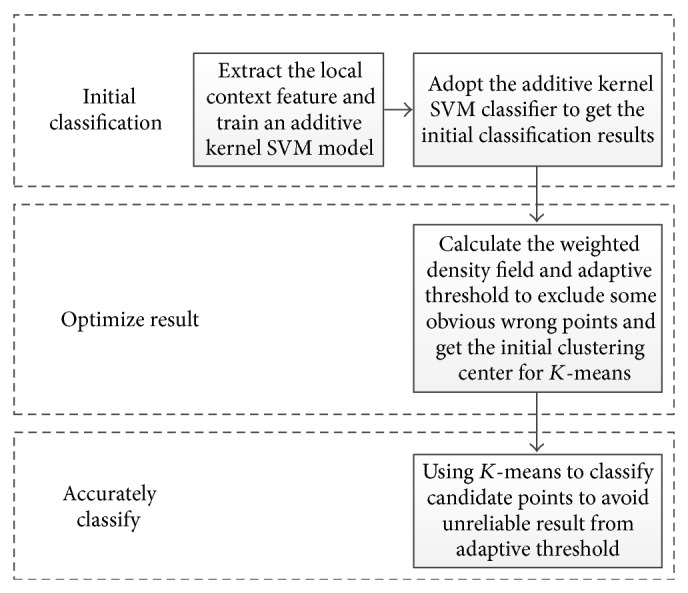
Flow of classification.

The flow of classification procedure can be integrated into three layers shown in Figure 5.

## 6. Experiments

The image data in the paper is from Sonos 7500 ultrasound image and the size of raw 3D image is 208 × 160 × 144. Data is acquired from 10 children who are from 9 to 12 years old. The cardiac cycle has 9 to 24 frames. Experiment 1 shows how to confirm the sampling window of local context feature and how to choose the size of weighted template. Experiment 2 compares the 3 ∗ 3 average sampling and direct sampling result in the heavy noise ultrasound images. Experiment 3 compares the result of this algorithm and manual selected points which are selected by professional doctors. Experiment 4 shows various results using different kernel functions.

### 6.1. Experiment 1

**Figure 6:**
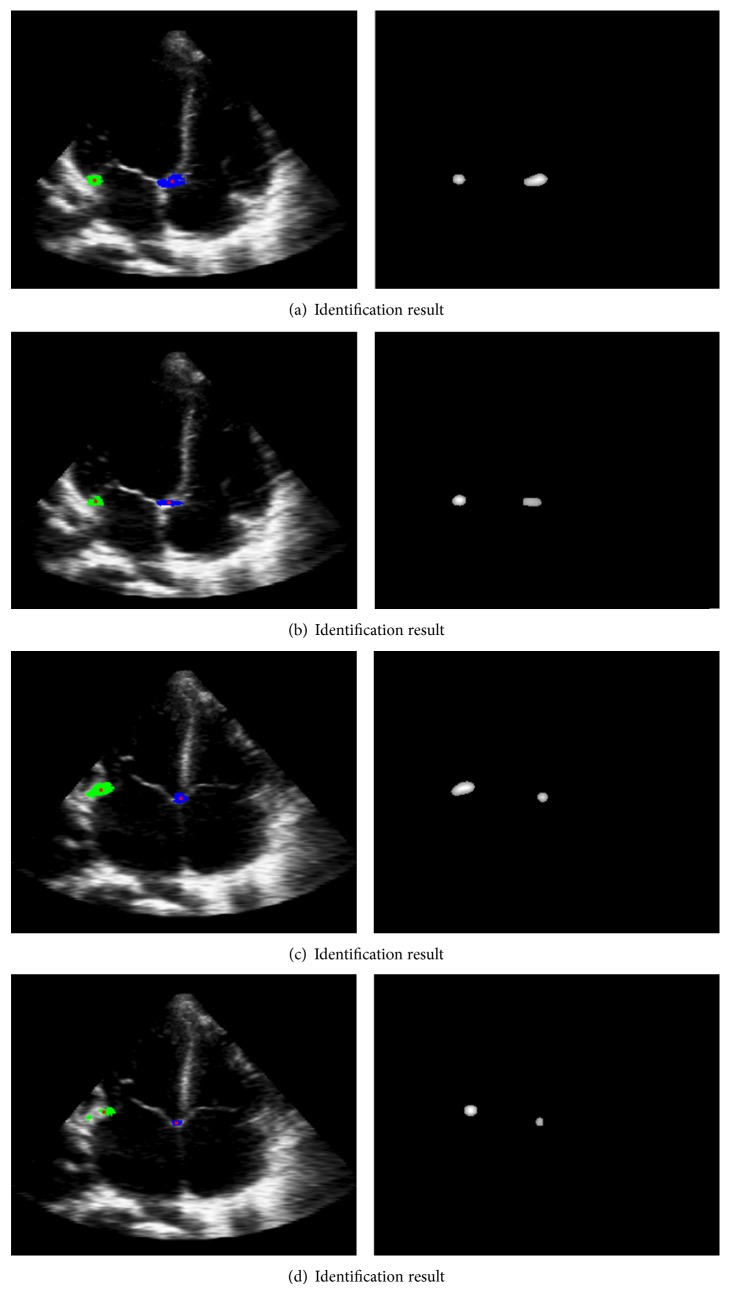
Identification results by different parameters.

Local context feature is to sample local structure of heart tissue. The specific size of sampling window and the size of weighted template are from experiments. And the experiment results show that different size has little influence to the results.  Figure 6(a) is the result of parameters as {1,3, 5,9, 13,17,23,29} in local context feature and Figure 6(b) is the result of parameters as {1,2, 4,8, 12,15,22}.  Figure 6(c) is the result of weighted template whose size is set to 10 and Figure 6(d) is the result of weighted template whose size is set to 8.

### 6.2. Experiment 2

**Figure 7:**
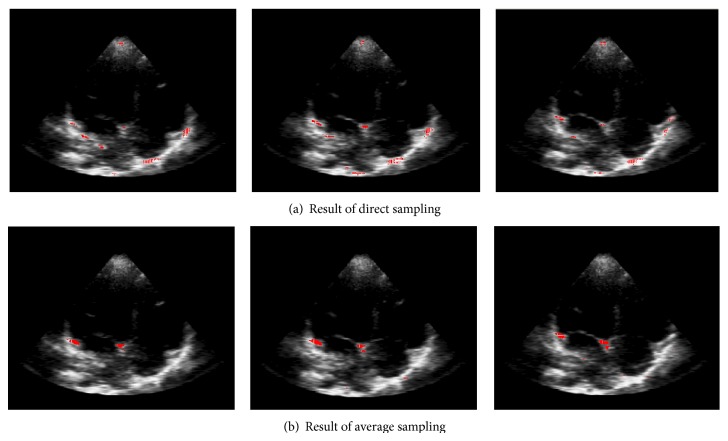
The classification result of different sample mode.

Figure 7(a) shows the result of direct sampling method, and Figure 7(b) shows the result of 3 ∗ 3 average sampling. The experiment shows that the result of 3 ∗ 3 average sampling is better than direct sampling. Additionally, we can see that heavy noise and low resolution in ultrasound image affect the result seriously. This denotes that the regular image detection methods cannot be used in medical images. HOG or SIFT feature which detect the corner points also cannot be used in medical images especially ultrasound images.

### 6.3. Experiment 3

Table 1 shows the mean and variance of our results compared to manual select points. This result is obtained from cardiac cycle of 10 patients. Our mean error can be control in nearly 0.96 mm which is acceptable in medical diagnosis.

### 6.4. Experiment 4

**Figure 8:**
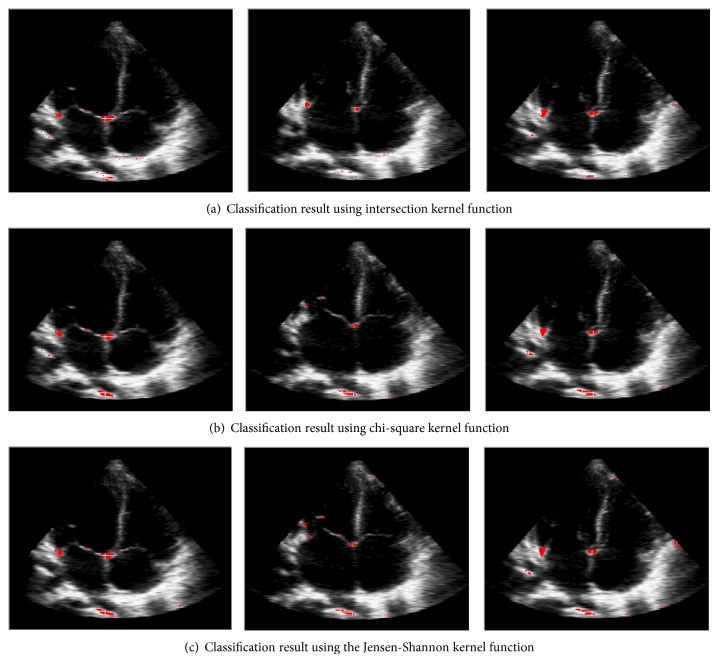
The classification result of different kernel function.

Figure 8 shows various results using different kernel functions. In this figure, we can conclude that the choice of kernel function is very important for final result. Furthermore, we can see that intersection histogram kernel which matches the local context feature gets a more accurate classification result.

## 7. Conclusions

This paper introduced a hinge point of mitral annulus identification method using additive kernel SVM classifier and local context feature. Due to the classification errors, we design a weighted template to exclude the obvious wrong points. After refining the result, the mean and variance of error between automatic and manual result are controlled in 0.96 ± 1.04 mm. From the experiments, it is demonstrated that this algorithm can accurately locate the hinge point of mitral valve. For the fast feature extraction and accelerated classification procedure, this algorithm can be used in real-time applications.

## Figures and Tables

**Table 1:** Errors between our segmentation method and manual segmentation results.

	Septal				Lateral			
	*x*-axis		*y*-axis		*x*-axis		*y*-axis	
	Mean	Variance	Mean	Variance	Mean	Variance	Mean	Variance
mm	0.96	0.907	1.12	0.69	1.34	1.39	0.75	0.48
